# Myoepithelial cell-driven acini contraction in response to oxytocin receptor stimulation is impaired in lacrimal glands of Sjögren’s syndrome animal models

**DOI:** 10.1038/s41598-018-28227-x

**Published:** 2018-07-02

**Authors:** Dillon Hawley, Xin Tang, Tatiana Zyrianova, Mihir Shah, Srikanth Janga, Alexandra Letourneau, Martin Schicht, Friedrich Paulsen, Sarah Hamm-Alvarez, Helen P. Makarenkova, Driss Zoukhri

**Affiliations:** 1 Depratement of Comprehensive Care, Tufts University School of Dental Medicine, Boston, MA USA; 2 Department of Molecular Medicine, The Scripps Research Institute, La Jolla, CA USA; 3 Department of Ophthalmology, USC Roski Eye Institute, Keck School of Medicine of University of Southern California, Los Angeles, CA USA; 4 Department of Anatomy II, Friedrich Alexander University Erlangen-Nürnberg, Erlangen, Germany; 5 University of Southern California School of Pharmacy, Los Angeles, CA USA; 6 Department of Ophthalmology, Tufts University School of Medicine, Boston, MA USA

## Abstract

The purpose of the present studies was to investigate the impact of chronic inflammation of the lacrimal gland, as occurs in Sjögren’s syndrome, on the morphology and function of myoepithelial cells (MECs). In spite of the importance of MECs for lacrimal gland function, the effect of inflammation on MECs has not been well defined. We studied changes in MEC structure and function in two animal models of aqueous deficient dry eye, NOD and MRL/lpr mice. We found a statistically significant reduction in the size of MECs in diseased compared to control lacrimal glands. We also found that oxytocin receptor was highly expressed in MECs of mouse and human lacrimal glands and that its expression was strongly reduced in diseased glands. Furthermore, we found a significant decrease in the amount of two MEC contractile proteins, **α**-smooth muscle actin (SMA) and calponin. Finally, oxytocin-mediated contraction was impaired in lacrimal gland acini from diseased glands. We conclude that chronic inflammation of the lacrimal gland leads to a substantial thinning of MECs, down-regulation of contractile proteins and oxytocin receptor expression, and therefore impaired acini contraction. This is the first study highlighting the role of oxytocin mediated MEC contraction on lacrimal gland function.

## Introduction

The non-keratinized epithelia of the ocular surface are constantly challenged by environmental insults such as smoke, dust, or airborne pathogens. Tears are the sole physical protective barrier for the ocular surface and contain mucins and lipids which sandwich a large aqueous layer containing water, electrolytes and proteins^1^. Mucins are secreted by the conjunctival goblet cells^2^, the meibomian glands secrete the lipid components^3^ and the lacrimal gland secretes the aqueous layer of the tear film^4^. Production of tears in insufficient quantity or of inadequate quality results in constant irritation of the ocular surface leading to dry eye disease also referred to as keratoconjunctivitis sicca (KCS)^5^. Patients with KCS can experience intense pain due to eye irritation, gritty/scratchy feeling in the eyes, blurry vision and light sensitivity^5^. Dry eye disease secondary to lacrimal gland deficiency is referred to as aqueous-deficient type of dry eye and can occur as a result of autoimmunity, such as Sjögren’s syndrome^5^. Dry eye disease also occurring in the absence of an underlying autoimmune disease is most prevalent in people over the age of 50 and post-menopausal women^6–9^.

Sjögren’s syndrome is the leading cause of aqueous-deficient dry eye and affects an estimated 1–4 million North Americans, mostly women^10^. Sjögren’s syndrome can present itself as a primary disorder affecting mainly the lacrimal and salivary glands, or it can be secondary to another autoimmune disease, such as rheumatoid arthritis, systemic lupus erythematosus, or systemic sclerosis^11^. The etiology of Sjögren’s syndrome, although still ill-defined, is thought to be multifactorial, involving viral, neural, genetic, and environmental factors^10–15^. The hallmarks of Sjögren’s syndrome are the lymphocytic infiltrates, which organize into foci within the lacrimal and salivary glands and the production of autoantibodies to both organ-specific and non-organ-specific autoantigens^11^.

The lacrimal gland secretory units are made up of acini and myoepithelial cells (MECs). The acinar cells are highly polarized with tight junctions creating apical and basolateral membranes as well as segregating ion channels therefore allowing for unidirectional secretion of the primary lacrimal gland fluid into the lumens^16^. MECs form a functional network around the acinar and ductal epithelial cells separating them from the basement membrane and mesenchymal stromal cells^17–20^. MECs have been shown to express muscarinic and purinergic receptors and therefore are able to respond to neural stimuli^21–23^. In addition, MECs synthesize components of the basement membrane and secrete growth factors and are thought to be able to contract to expel fluid and proteins from the acini^24–26^. MECs express a number of contractile proteins, such α-smooth muscle actin (SMA) and calponin, as well as epithelial markers such as keratins (keratin 5 and keratin 14)^20^.

MECs are best studied in the mammary gland where it was shown that their contraction is crucial for milk production and that knockout of SMA expression leads to impaired milk secretion^27^. In addition, MEC contraction in the mammary gland is controlled by the neuropeptide oxytocin and activation of the oxytocin receptor (OXTR)^28^. Despite their potential critical role in lacrimal gland secretion, very little is known about MEC contraction in this tissue, nor the impact of chronic inflammation of the lacrimal gland on these cells.

Several studies reported abnormal basement membrane and extracellular matrix (ECM) production in Sjögren’s syndrome lacrimal and salivary glands^29–33^. Since MECs are responsible for production of components of the ECM and basement membrane^24–26^, we hypothesized that chronic inflammation of the lacrimal gland as occurs in Sjögren’s syndrome, affects MEC morphology and function. To test this hypothesis, we used two well-studied animal models of human Sjögren’s syndrome to determine the impact of chronic inflammation on lacrimal gland MECs. Our data show that MECs of chronically inflamed lacrimal glands express less contractile proteins and oxytocin receptor when compared to those from control non-inflamed glands. Moreover, acini of diseased lacrimal glands were not able to contract in response to oxytocin stimulation, suggesting that MEC and acini contractile function is strongly affected by inflammation.

## Results

MRL/lpr and NOD mice are excellent models that mimic different pathologies of Sjögren’s syndrome’s autoimmune driven lacrimal gland deficiency^34,35^. Both strains develop spontaneous, age- and sex**-**dependent infiltration of the lacrimal gland by auto-reactive lymphocytes, which lead to loss of lacrimal gland epithelial secretory components^34,36^. In contrast, lacrimal glands from age- and sex-matched control animals do not show any signs of inflammation. As we previously reported^37^, lacrimal gland inflammation is more severe in NOD mice when compared to the MRL/lpr mice.

**Figure 1:**
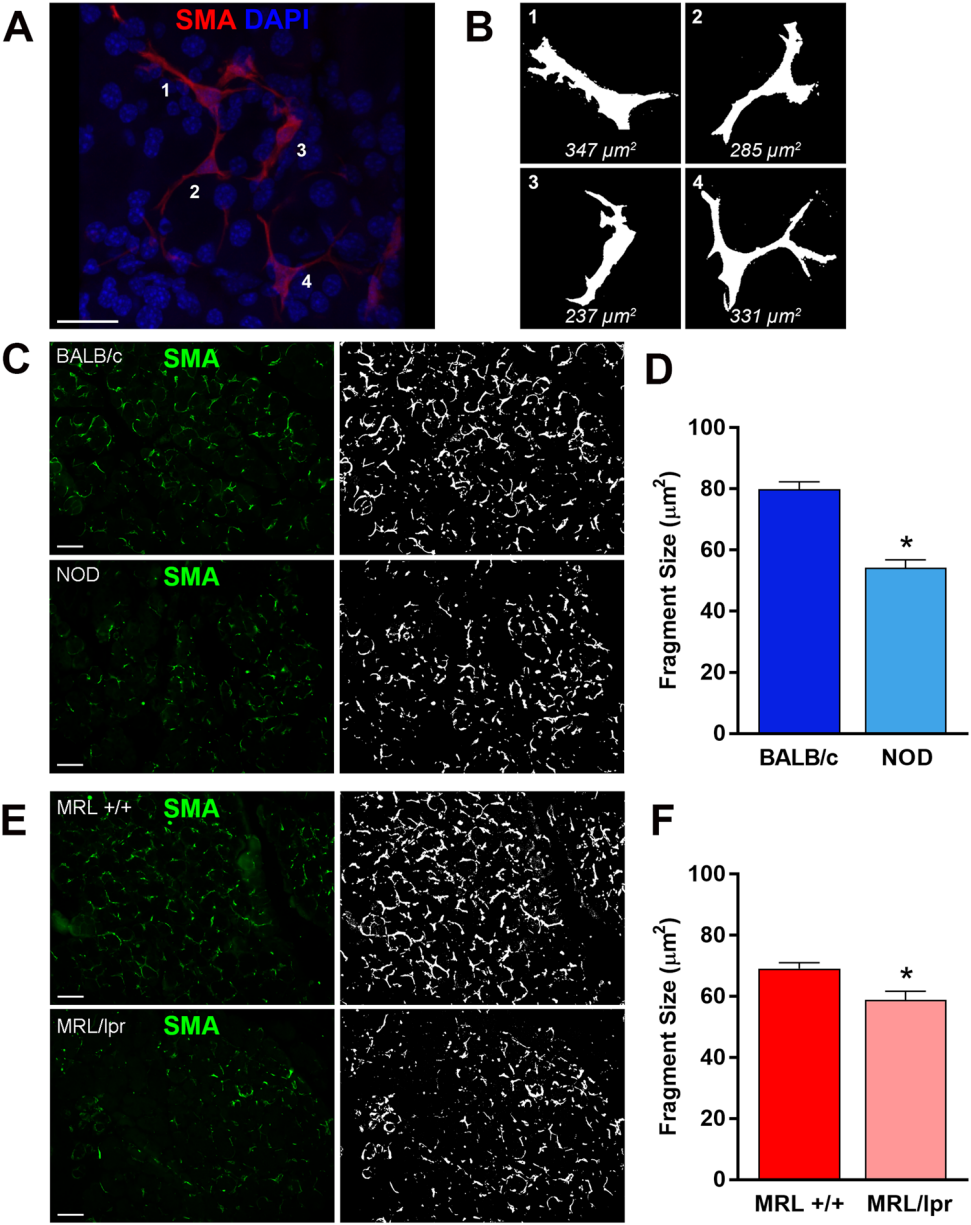
Determination of MEC size in healthy and diseased lacrimal glands. (**A**) Lacrimal glands from BALB/c mice were stained with an antibody against α-smooth muscle actin (SMA) and imaged by confocal microscopy to visualize whole MECs. (**B**) ImageJ was used to create masks of MECs in the normal lacrimal gland to generate the representative sizes. The same method was then used to compare MEC size, using SMA staining (green; **C**,**E**) in lacrimal glands of diseased and healthy mice. MEC staining was masked using ImageJ (white; **C**,**E**) and quantified to determine the average size of the stained fragments to compare NOD and BALB/c (**C**,**D**) or MRL/lpr and MRL +/+ (**E**,**F**). Star denotes statistically significant difference compared to control; n = 6 for MRL/lpr mice and n = 5 for all other groups; scale bar represents 25 µm (**A**) and 50 µm (**C**,**E**).

**Figure 2:**
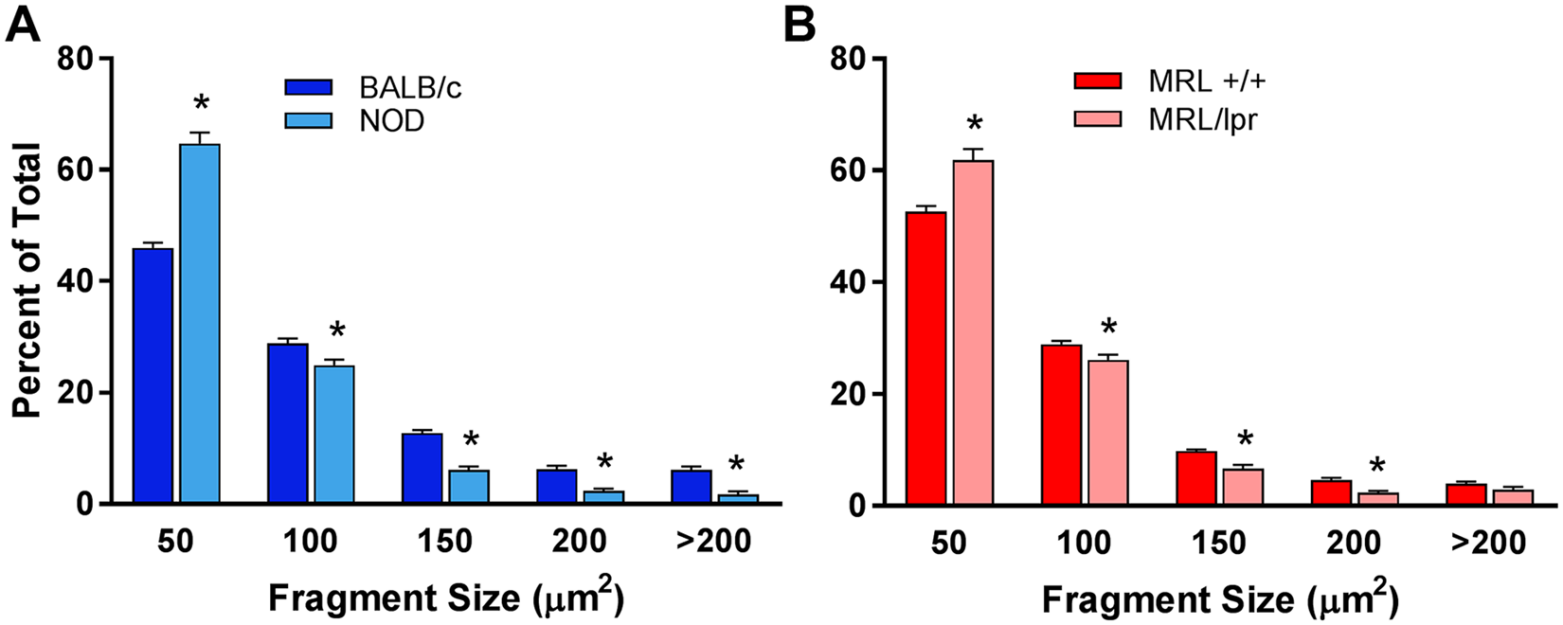
Distribution of MEC size in healthy and diseased lacrimal glands. The distribution of the α-smooth muscle actin staining was plotted to identify differences in distribution of fragment size. Diseased mice (NOD and MRL/lpr) showed an increase in small fragments and a decrease in large fragments compared to healthy mice (BALB/c and MRL +/+). Star denotes statistically significant difference compared to control; n = 6 for MRL/lpr mice and n = 5 for the other groups.

Similar to other exocrine gland MECs, lacrimal gland MECs have a small (around 10–15 μm) cell body and several long, often branched processes, which causes the MEC to occupy a large area^20,38^. To address the impact of inflammation on MECs morphology, we developed a method to estimate the size of α-smooth muscle actin (SMA) stained lacrimal gland MECs (Fig. 1B) (see Materials and Methods section). Lacrimal gland sections from diseased and control animals were stained with an antibody against SMA and MEC size was measured as described in the Methods section. The experimental data reported in Fig. 1C–F show that when compared to lacrimal glands from control animals (BALB/c and MRL +/+), there was a statistically significant 32.1% and 14.8% reduction in MEC size in NOD and MRL/lpr lacrimal glands; respectively. The photomicrographs in Fig. 1C,E depict representative SMA staining in control and diseased lacrimal glands and the plots in Fig. 1D,F show averaged data obtained using lacrimal glands from 5 different animals in each group (6 animals for MRL/lpr). We also looked at the distribution of MEC sizes (using the method described in Fig. 1) and found that diseased mice had significantly more small sized cells/processes (<50 µm^2^) compared to control mice (Fig. 2). Lacrimal glands from diseased mice had also a significant reduction of clusters containing larger sized cells/processes (from 100 to >200 µm^2^, Fig. 2), which we believe is representative of full-bodied cells with long processes (see for example the image in Fig. 1B, panel 1).

**Figure 3:**
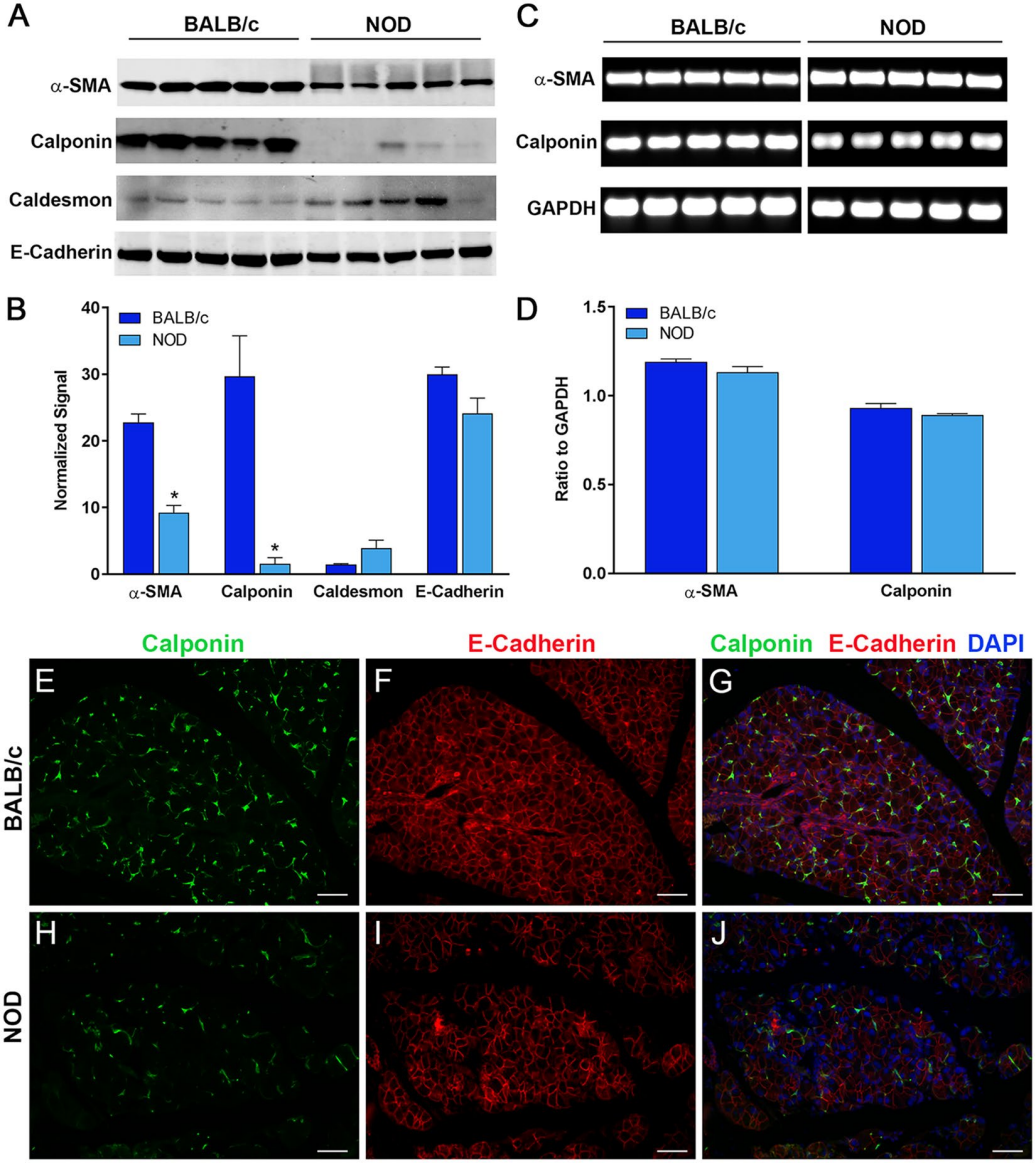
Decrease in smooth muscle associated proteins in NOD mice. Total proteins and RNA were extracted from the lacrimal glands of NOD and BALB/c mice and processed for (**A**) SDS-PAGE / Western blotting and (**C**) RT-PCR analysis, respectively. (**B**) Western blot staining was normalized to REVERT^TM^ total protein stain showing significant decreases in α-smooth muscle actin and calponin but not caldesmon and e-cadherin protein expression in NOD LGs compared to control BALB/c LGs. (**D**) RT-PCR was normalized to internal GAPDH controls (representative shown) but no difference in mRNA levels was detected. Immunofluorescent double-staining of healthy BALB/c (**E–G**) and diseased NOD (H-J) LGs for calponin and E-cadherin show decreased staining of calponin in diseased NOD LGs. Star denotes statistically significant difference compared to control; n = 5 for each group; scale bar represents 50 µm (**E**–**J**). For protein blots NOD and BALB/c samples were run and cropped from the same gel and for PCR each gene of interest and control gene (GAPDH) were run and cropped from the same gel; for accurate standardization. Full blots are provided in the supplementary data file.

In another series of experiments, we determined the level of expression of several MEC contractile proteins (SMA, calponin, and caldesmon) using Western blotting of NOD and control lacrimal glands. Since we only had paraffin blocks but no cell lysates from MRL/lpr and MRL +/+ mice lacrimal glands, only cell lysates from NOD mice lacrimal glands were used. Calponin and caldesmon are two thin filament-binding proteins found in smooth muscle that have both been attributed a role in modulating the interaction of actin and myosin. As shown in Fig. 3A, lacrimal gland samples from NOD mice had down-regulated SMA and calponin protein expression compared to samples from control BALB/c mice. Interestingly the caldesmon protein level tended to be higher in NOD samples, however not significantly (p = 0.11; Fig. 3A). In contrast, the amount of the epithelial cells marker E-cadherin was only slightly decreased in NOD lacrimal glands compared to control ones, suggesting that reduction of contractile apparatus proteins was not due to a decrease of lacrimal gland epithelial component (Fig. 3A). To ensure equal loading for all samples total protein level in each sample was determined on PVDF membranes by staining with REVERT, a validated method for normalization of Western blots^39–41^. The Li-COR Odyssey software was employed to quantify the amount of proteins in each sample on the REVERT stained membranes and we used these values to normalize the amount of SMA, calponin, caldesmon and E-cadherin staining obtained by Western blotting. The data reported in Fig. 3B show that there was a statistically significant 59.7% and 94.8% decrease in the amount of SMA and calponin proteins, respectively, in lacrimal gland samples prepared from NOD mice when compared to lacrimal glands from control BALB/c mice. Changes in expression levels of caldesmon and E-cadherin in NOD lacrimal glands compared to control glands were not statistically significant (Fig. 3B).

The decreased expression of calponin protein was confirmed using immunostaining. As shown in Fig. 3E–J, calponin staining was diminished in lacrimal gland samples from NOD mice (Fig. 3H) when compared to those from control BALB/c mice (Fig. 3E). The E-cadherin immunostaining, which outlines the acinar cells, was not altered in NOD samples (Fig. 3I) compared to BALB/c samples (Fig. 3F).

To determine if SMA and calponin expression is altered at the transcriptional level, we performed semi-quantitative RT-PCR experiments using RNA extracted from NOD and BALB/c lacrimal glands. As shown in Fig. 3C,D, the mRNA levels for either SMA or calponin were not significantly different between samples from NOD mice and BALB/c mice. These data suggest that the effect of inflammation on SMA and calponin expression occurs at a post-transcriptional level. Several studies have shown that during the inflammatory process, MMP-2^42^ and calpain^43^ can degrade calponin while others showed that caspase 3^44^ and the ubiquitin/proteasome pathway^45^ are involved in degradation of skeletal muscle proteins.

Taken together, these data suggest that chronic inflammation of the lacrimal gland leads to thinning of MEC processes and therefore an overall decrease in MEC size. We hypothesize that this decrease in MEC size (i.e., thinning of cell processes) and the decreased expression of SMA and calponin would reduce their contractile properties and therefore lead to reduction of the secretory function of the lacrimal gland.

Contraction of many smooth muscle cell types, including mammary gland MECs, is induced by oxytocin^28,46^. Oxytocin is a neurotransmitter and a hormone known to control smooth muscle cell contraction in multiple tissues^47^. In smooth muscle cells, activation of the oxytocin receptor leads to a transient elevation in intracellular calcium levels resulting in cell contraction^47,48^.

**Figure 4:**
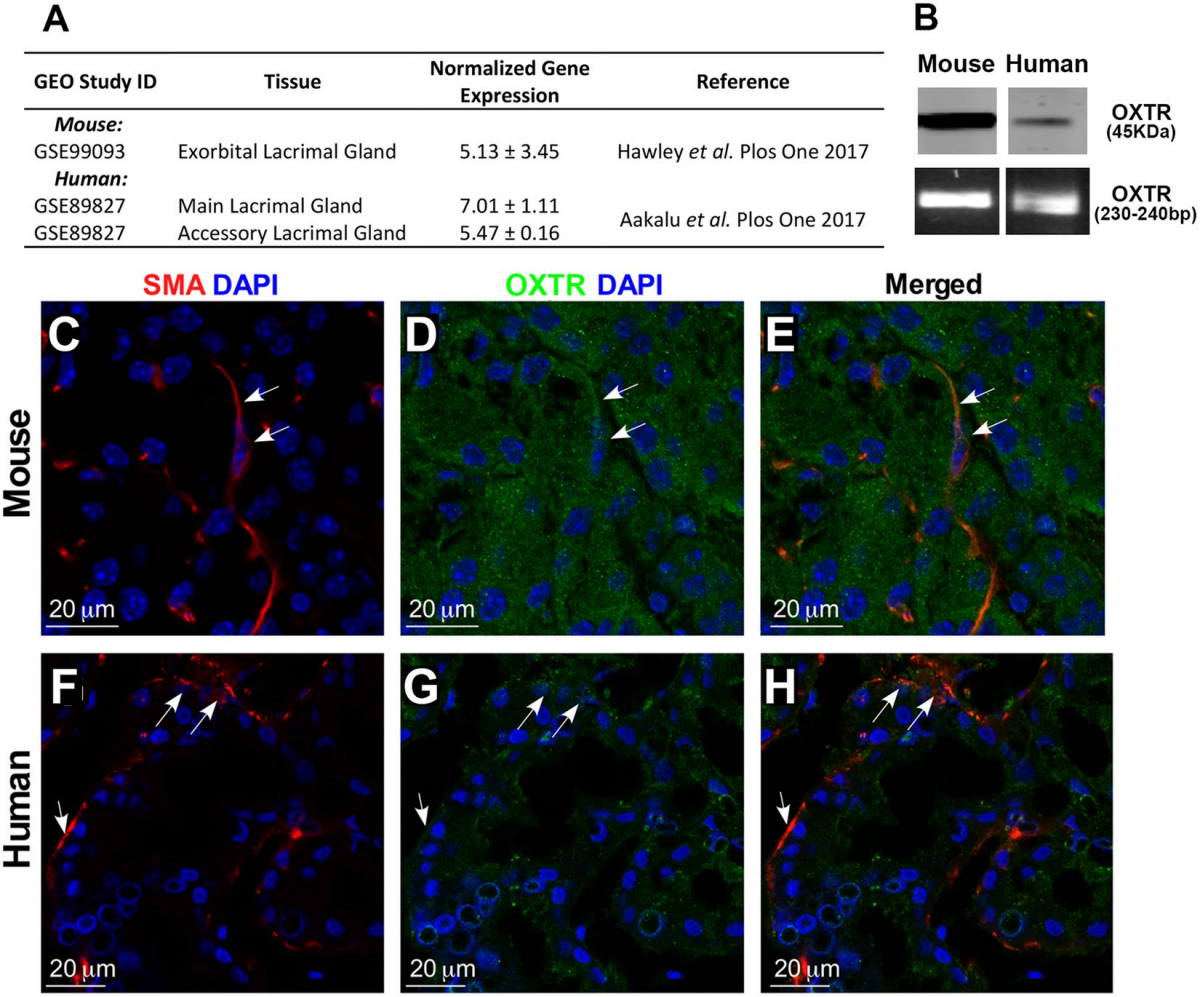
Oxytocin Receptor expression in normal human and mouse lacrimal glands. (**A**) Compiled publically available transcriptomic analyses of human and mouse lacrimal glands indicate expression of the oxytocin receptor gene. Using Western blottingand RT-PCR (**B**), and immunofluorescent staining of normal mouse (**C–E**) and human (**F–H**) lacrimal glands confirmed expression of the oxytocin receptor in the lacrimal gland. For protein and PCR blots mouse and human samples were run and cropped from the same gel for accurate standardization.

**Figure 5:**
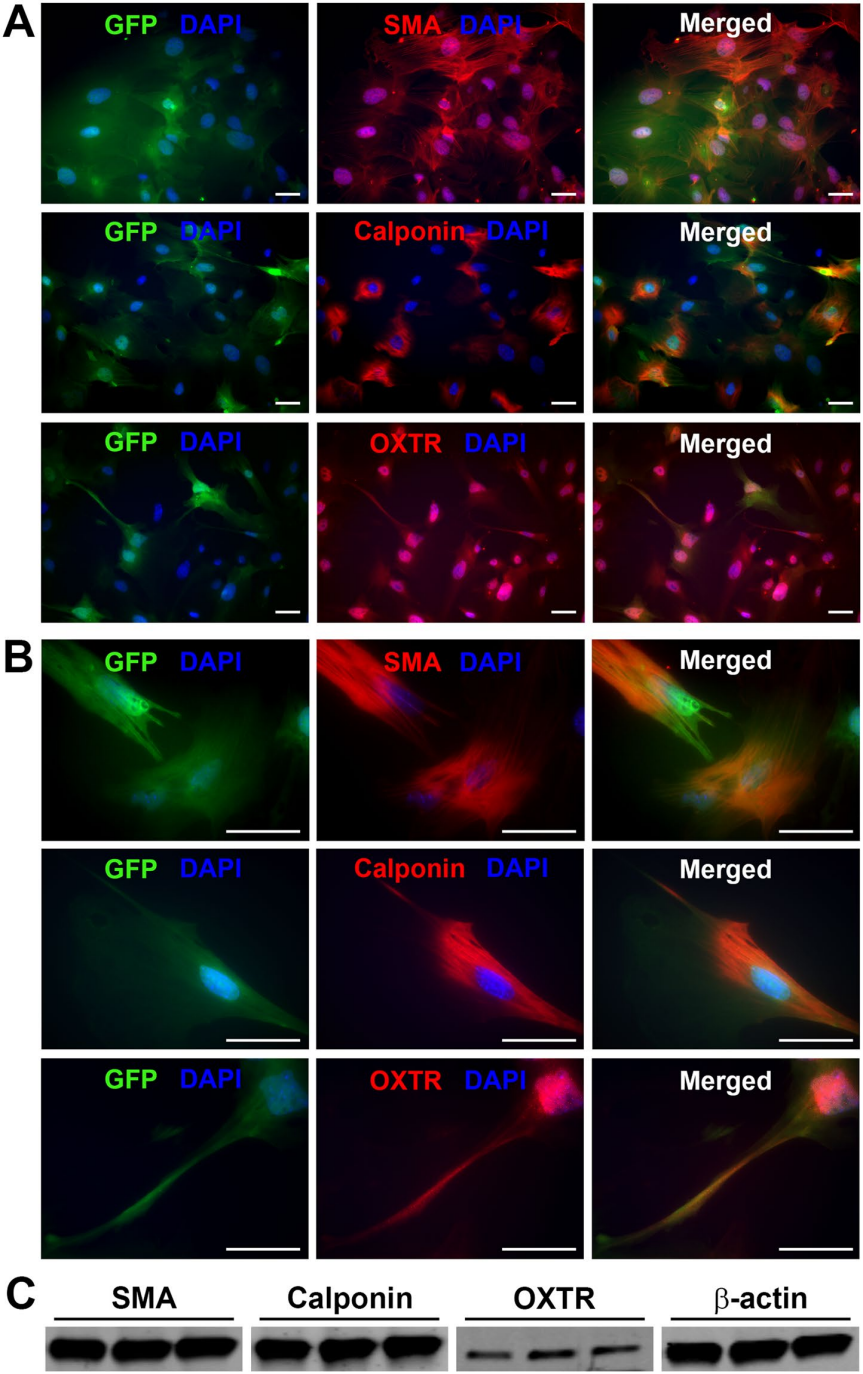
Expression of Oxytocin receptor and smooth muscle proteins in isolated MECs. Lacrimal glands from SMA-GFP mice were digested and single cell isolates were used to sort GFP-expressing MECs. We used immunofluorescence (**A**,**B**) staining for SMA, calponin, and oxytocin receptor, of sorted MECs, to confirm expression and co-localization in GFP-expressing cells. (**C**) Proteins were isolated from sorted MECs and used for Western blotting to confirm the expression of SMA, calponin, and oxytocin receptor in lacrimal gland MECs. Cells stained with only the secondary antibody were used as a negative control (not shown). Scale bar represents 50 µm (**B**,**C**).

To determine whether lacrimal gland MECs and/or acinar cells have the ability to respond to oxytocin stimulation we interrogated RNAseq data^49^ and found that oxytocin receptor is expressed in the murine lacrimal gland (Fig. 4A). Published microarray data also reported the expression of this receptor in human main and accessory lacrimal glands (Fig. 4A)^50^. Western blotting, RT-PCR analysis, and immunostaining with antibodies against the oxytocin receptor showed that it is expressed in both human and mouse lacrimal glands (Fig. 4B–H). Immunostaining shows that the oxytocin receptor is present on both acini and MECs (Fig. 4). To further confirm that the oxytocin receptor is expressed in MECs, we sorted GFP labeled MECs from SMA-GFP mice lacrimal glands and used them for immunostaining and Western blotting. As shown in Fig. 5, GFP-labeled MEC express the oxytocin receptor as demonstrated by immunostaining (5 A and B) as well as Western blotting (5 C). We also stained these cells for SMA and calponin to ensure that they are MECs (Fig. 5A,B).

These findings suggest that the mouse and human lacrimal gland may be responsive to oxytocin stimulation.

**Figure 6:**
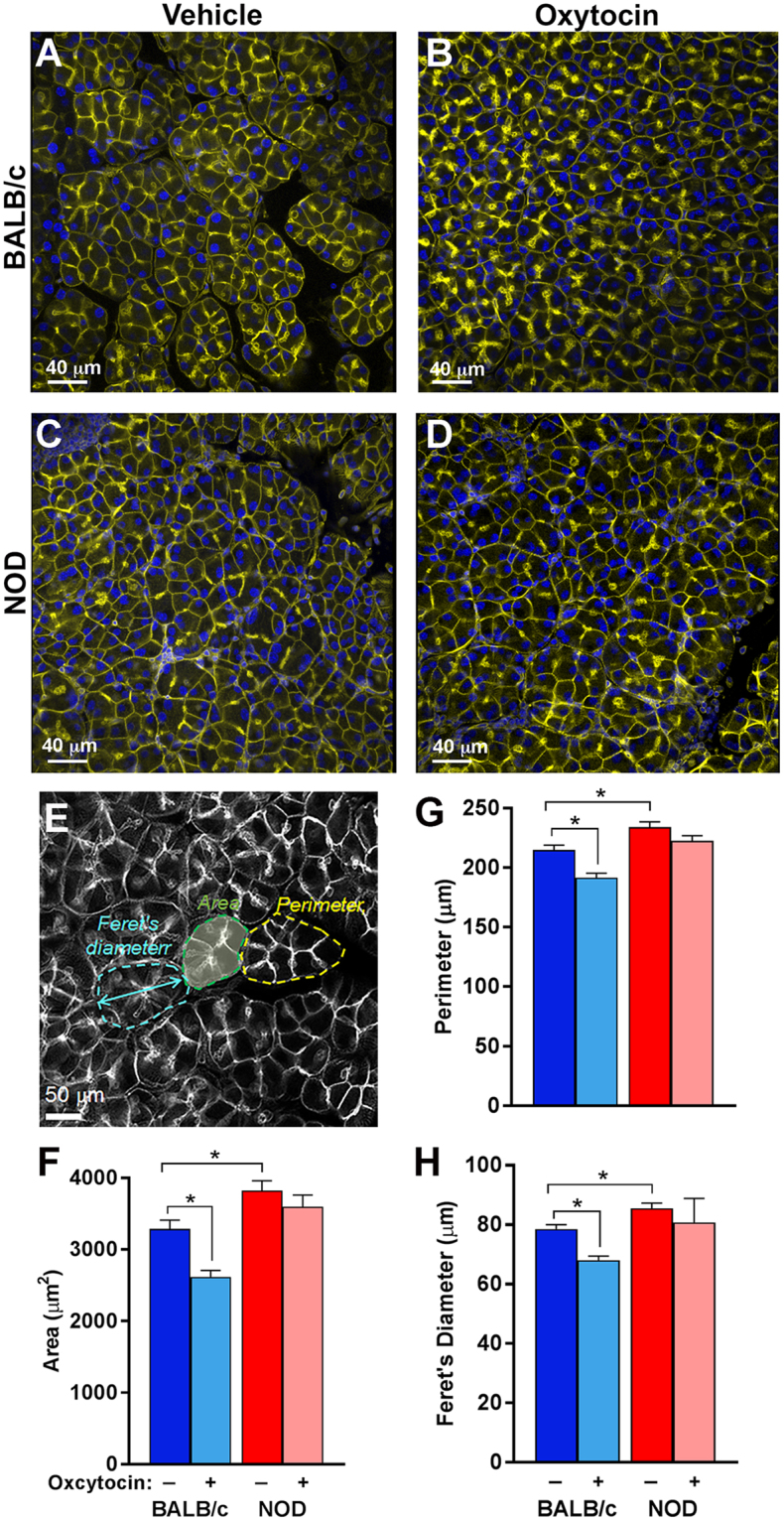
Decrease in contraction of MECs from NOD mice. Lobules prepared from the lacrimal glands of NOD and BALB/c mice were treated with oxytocin or PBS (vehicle) to stimulate MECs contraction. (**A**,**C**) show the basal acinus size in non-stimulated lacrimal glands from BALB/c and NOD mice respectively, while (**B**,**D**) show the acinus size after stimulation with 5 nM oxytocin for these mice; respectively. The acinus size was measured using 3 metrics, as depicted in (**E**). Acinus size was compared using (**F**) area, (**G**) perimeter, and (**H**) Feret’s diameter. Star indicates statistically significant difference compared to the indicated group; n = 3 per group.

We tested this hypothesis by measuring oxytocin-mediated contraction of lacrimal gland acini as described in mammary glands^51^ (also see Materials and Methods). In these experiments, acini contraction was stimulated with oxytocin and the size of acini was compared to control glands treated with PBS (vehicle). We found that oxytocin stimulation of control lacrimal glands resulted in substantial contraction of lacrimal gland acini while diseased acini of NOD mice did not contract (Fig. 6). To quantify acini contraction we measured three different parameters of lacrimal gland acinus size: area, perimeter, and Feret’s diameter (a measurement of the maximum diameter) as depicted in Fig. 6E.

The photomicrographs in Fig. 6(A–D) depict phalloidin stained acini from stimulated and vehicle treated lacrimal glands of NOD and BALB/c mice. In control BALB/c lacrimal glands treated with oxytocin there was a statistically significant reduction in acini area, perimeter and Feret’s diameter compared to lacrimal glands treated with vehicle (Fig. 6A,B): the acinus area, perimeter and Feret’s diameter were reduced by 20, 11, and 13% following oxytocin stimulation (Fig. 6F–H). In contrast lacrimal glands obtained from NOD mice showed no visible reduction in acinar size (Fig. 6C and D); there was no significant reduction in acinus area, perimeter or Feret’s diameter (Fig. 6F–H). Moreover, comparing the acinar sizes in unstimulated BALB/c and NOD mouse lacrimal glands showed that acinus perimeter (Fig. 6G) and diameter (Fig. 6H) were significantly longer (9% and 9%, respectively) than in control BALB/c glands. In addition average acinus area (Fig. 6F) was 16% significantly bigger in NOD versus BALB/c mouse lacrimal glands.

Taken together these data suggest that in chronically inflamed lacrimal glands, the contractile functions of MECs and acini are significantly altered.

**Figure 7:**
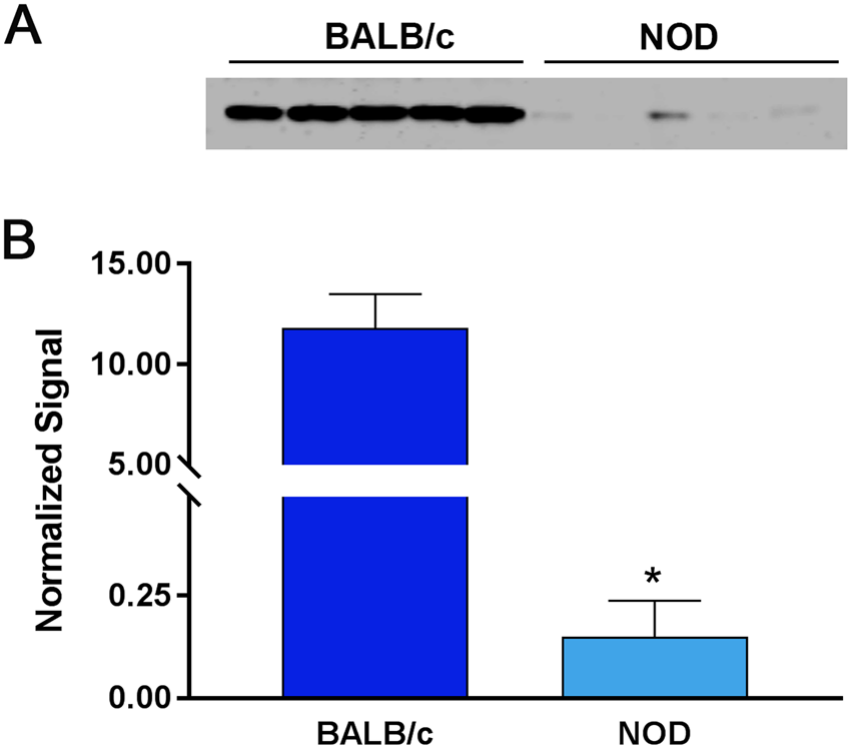
Decrease in oxytocin receptor expression in MECs of NOD mice. (**A**) Total protein from healthy BALB/c and diseased NOD LGs were processed for SDS-PAGE / Western blotting. (**B**) Western blots were normalized to REVERT^TM^ total protein stain showing a significant reduction in oxytocin receptor in NOD LGs compared to BALB/c. Star indicates statistically significant difference compared to the control group; n = 5 per group. For protein blots NOD and BALB/c samples were run and cropped from the same gel; for accurate standardization. Full blots are provided in the supplementary data file.

Since NOD lacrimal gland acini and MECs were irresponsive to oxytocin stimulation, we hypothesized that oxytocin receptor expression may be reduced in NOD lacrimal glands. To test this, we performed Western blotting using lacrimal glands obtained from BALB/c and NOD mice. The data shows (Fig. 7A and B) a severe 98.7% reduction of oxytocin receptor protein expression in diseased lacrimal glands compared to control glands.

Taken together, these data suggest that the partial loss of oxytocin receptor expression coupled with MEC thinning is responsible for the decrease in tear secretion from diseased lacrimal glands of NOD mice.

## Discussion

Our data show that chronic inflammation of the lacrimal gland in mouse models of Sjögren’s syndrome leads to thinning of the MECs and therefore impaired function of these cells in the lacrimal gland. This finding is supported by Hayashi *et al*.^52^ who conducted a study on the MECs of salivary glands from New Zealand Black/New Zealand White (NZB/NZWF_1_) mice, an animal model of Sjögren’s syndrome. The authors examined ultrastructural changes in the MECs within the inflammatory lymphocytic foci. Using electron microscopy, they reported a substantial destruction of the MECs and lysis of basement membranes^52^. In our study we concentrated on areas between the lymphocytic foci where acini still appear to be unaffected by the disease. However, we found substantial changes in the MECs morphology and contractile proteins expression in these relatively “healthy” areas of the lacrimal gland. The thinning of MECs has been also reported^53^ in the parotid glands of diabetic mice. Decreased expression of SMA as well as skeletal muscle actin-α and the water channel aquaporin 8 in parotid glands of diabetic mice was reported^53^. Another study^54^ on parotid glands from human patients with sialadenosis reported a major loss and thinning of the myofilament component of the MECs and significant acini enlargement. The last finding correlates with our study also showing enlargement of acini in diseased lacrimal glands.

In contrast, a recent study by Gervais *et al*.^55^ reported an increase in SMA expression in the submandibular glands from older 22-wk, but not 18-old NOD mice. The authors hypothesized that this result may reflect an undergoing process of epithelial mesenchymal transition (EMT) induced by severe inflammation in which ‘migratory’ epithelial cells and invading myofibroblasts and immune cells will express SMA. The same authors reported, however, a decrease in SMA staining level in minor salivary glands biopsies from Sjögren’s patients^55^. The findings in human biopsies are in accordance with our study.

Other studies show that SMA protein is critical for adequate contraction of MECs to occur^27^. Indeed, Haaksana *et al*. showed that in SMA deficient mice the contractile function of mammary gland MECs is significantly reduced^27^. Reduced contractile ability of MECs in diseased lacrimal glands upon stimulation with oxytocin could be explained by the reduction in SMA protein expression. The reduction of oxytocin receptor protein expression would further impair MECs function. Since the oxytocin receptor is also expressed in the acinar cells, its decreased expression in diseased glands would contribute to the loss of contractibility in response to oxytocin stimulation and the resulting decreased secretion of aqueous tear components.

It has been reported that inflammatory cytokine, especially IL-1β, down-regulate the expression of the oxytocin receptor in uterine smooth muscle^56,57^. The effect of IL-1β was shown to be both at the mRNA level as well as the oxytocin receptor protein level, although the molecular mechanisms were not described^57^. We have previously shown that the amount of IL-1β and its signaling receptor were significantly up-regulated in diseased lacrimal glands of MRL/lpr mice^58^ and hypothesize that this cytokine might be responsible for the down-regulation of the oxytocin receptor that we describe in the present study.

Smooth muscle cells, called pericytes, are present in the vasculature^59^. A recent study by Castro *et al*.^42^ reported that matrix metalloproteinase 2 (MMP2) may interact with calponin-1 in aortic vascular smooth muscle cells and that MMP2 mediated proteolysis of calponin-1 during endotoxemia may contribute to LPS-induced hypocontractility. In our recent study we also reported elevated activity of MMP2 in chronically inflamed lacrimal glands^60^ and showed that inhibition of MMP2 activity, *in vivo*, restored normal tears secretion. However, the mechanistic understanding of the effect of MMP2 inhibition on calponin-1 expression and MEC function needs further investigation.

In summary, our data show MEC thinning, due to decreased expression of the contractile proteins SMA and calponin, and significant loss of the oxytocin receptor which may contribute to impaired function of MECs in chronically inflamed lacrimal glands. This may be responsible for the primary decrease in tears secretion in Sjögren’s syndrome patients. Although future studies are needed to test this hypothesis, the recent study of Gervais *et al*.^55^ showing decreased SMA staining in minor salivary glands biopsies from Sjögren’s patients supports this idea.

## Materials and Methods

### Animals and treatments

All experiments described herein were performed in accordance with the Association for Research in Vision and Ophthalmology (ARVO) Statement for the Use of Animals in Ophthalmic and Vision Research and were approved by the Tufts Medical Center Animal Care and Use Committee, the University of Southern California Institutional Animal Care and Use Committee, the Scripps Research Institute Animal Care and Use Committee, and Regierung von Unterfranken (Friedrich Alexander University Erlangen-Nürnberg). MRL/MpJ-Fas <lpr>/J (MRL/lpr; female, 12 weeks old) and NOR/LtJ (NOD; male, 13 weeks old) mice and their respective age- and sex-matched MRL/MpJ (MRL +/+) and BALB/c control mice were purchased from the Jackson Laboratories (Bar Harbor, ME). We also used in-house bred, 12–13-week-old male NOD mice that were purchased from Taconic Biosciences (Hudson, NY). Animals were euthanized and the exorbital lacrimal glands were harvested and processed for paraffin embedding, total protein extraction, RNA extraction or stimulation with oxytocin. In other experiments, SMA-GFP mice (kind gift of Dr. Ivo Kalajzic), in which lacrimal gland MECs are labeled with GFP, were used to isolate MECs from the lacrimal gland by fluorescence-activated cell sorting (FACS).

### Acquisition of human tissue

Human lacrimal glands from three donors were obtained from Advanced Tissue Services (Phoenix, AZ). The study was reviewed by the Tufts Medical Center/Tufts University Health Sciences Institutional Review Board (IRB) and was determined to be exempt in accordance with 45 CFR.101(b)(4). Tissue (whole lacrimal gland) was preserved immediately in *RNAlater* (about 60 mL/gland) and shipped on ice overnight. Following acquisition lacrimal gland tissue was divided and processed for paraffin embedding, total protein extraction, or RNA extraction.

### Isolation and growth of lacrimal gland MECs

Lacrimal glands were excised from 3 week-old SMA-GFP mice and minced into small pieces for digestion. Tissue was then digested with 0.75 mL/lacrimal gland Collagenase digestion solution (464 U/mL Collagenase II (Worthington Biochemical, Lakewood, NJ) in DMEM containing 2 mM L-glutamine, 1% non-essential amino acids, 1% penicillin/streptomycin, and 8 U/mL DNAse I) for 50 minutes at 37 °C and 100 RPM. During the digestion the tissue was broken up by triturating every 15 minutes with increasingly smaller pipette tips. After digestion the digested tissue was filtered through a 100 µm mesh and washed two times with Ca^2+^ and Mg^2+^ -free phosphate-buffered saline (PBS, 137 mM NaCl, 2.7 mM KCl, 6.5 mM Na_2_HPO_4_, and 1.5 mM KH_2_PO_4_ at pH 7.2) +2 mM EDTA to stop Collagenase digestion. Next digested tissue was incubated with room temperature TrypLE Express (Invitrogen, Carlsbad, CA) for 2 minutes at 37 °C to isolate single cells. Then TrypLE Express was diluted with DMEM containing 1 U/mL DNAse I for 3 minutes followed by washing with DMEM + 10% fetal bovine serum (FBS) to inactive any residual Trypsin activity. Finally isolated single cells were re-suspended in MEC culture media (RPMI 1640 + 10% FBS + 2 mM L-glutamine + 1% penicillin/streptomycin).

GFP + cells were sorted using a FACS Aria cell sorter (Becton Dickinson, Franklin Lakes, NJ) and plated at a density of ~15,000 cells/cm^2^ on tissue culture dishes coated with 3.5 µg/cm^2^ CellTak (Corning, Corning, NY) following the manufacturer’s instructions. Cells were grown in MEC media for 1 week and used for immunofluorescence staining or protein was isolated for Western Blotting.

### Immunofluorescence staining

Lacrimal glands and cells isolated from SMA-GFP mice (grown on 8-chamber slides (Fisher Scientific, Hampton, NH)) were fixed in 4% methanol-free formaldehyde prepared in PBS overnight at 4 °C or for 20 minutes at room temperature, respectively, followed by washing with PBS. Lacrimal gland tissue was embedded in paraffin and sectioned onto slides (6 µm). Tissue sections were then dried at 55 °C and de-paraffinzed in xylenes followed by re-hydration in graded ethanol solutions. Tissue sections were next subjected to heat-mediated antigen retrieval using VectorLabs un-masking solution (Burlingame, MA). Next slides containing tissue and cells were permeabilized using 0.1% Triton X-100 for 10 minutes at room temperature. Subsequently, non-specific binding sites were then blocked using 10% normal donkey serum (Jackson ImmunoResearch Laboratories, Westgrove, PA) for 1 hour at room temperature. The slides were then incubated overnight at 4 °C with the appropriate primary antibody (Table 1). Slides without primary antibody were included as negative controls. Next, slides were incubated with a compatible secondary antibody (Table 1) for 1 hour at room temperature. After each antibody incubation slides were washed with PBS containing 0.05% Tween-20. Finally, slides were covered with VectaShield Antifade mounting medium containing DAPI (VectorLabs) to counterstain nuclei.

**Table 1:** Antibodies used in immunohistochemistry and western blotting.

Target	Host Species/Conjugate	Vendor	Dilution	
***Primary Antibodies:***				
α-SMA	Rabbit (IgG)	Abcam (Cat#: ab5694)	WB:	1:400
			Immuno:	1:100
Calponin	Rabbit (IgG)	Abcam (Cat#: ab46794)	WB:	1:2000
			Immuno:	1:200
Caldesmon	Mouse (IgM)	Santa Cruz (Cat#: sc-271222)	WB:	1:500
			Immuno:	N/A
E-cadherin	Mouse (IgG)	BD Trans Labs (Cat#: 610181)	WB:	1:2500
			Immuno:	1:300
Oxytocin Receptor	Rabbit (IgG)	Abcam (Cat#: ab181077)	WB:	1:1000
			Immuno:	1:100
***Secondary Antibodies:***				
Rabbit IgG	AlexaFluor 680	Invitrogen (Cat#: A-21076)	WB:	1:5000
	AlexaFluor 488	Jackson Labs (Cat#: 711-545-152)	Immuno:	1:100
	Cy3	Jackson Labs (Cat#: 711-185-152)	Immuno:	1:100
Mouse IgM	HRP	Santa Cruz (Cat#: sc-516102)	Immuno:	1:1000
Mouse IgG	IRDye 800CW	Li-COR (Cat#: 925-32210)	WB:	1:5000
	Cy3	Jackson Labs (Cat#: 715-165-150)	Immuno:	1:100

### Image acquisition and analysis

All images were captured using the same imaging parameters with a SPOT RT Color camera (version 2.2.1; Diagnostic Instruments, Sterling Heights, MI). Five images were randomly captured from each slide. For diseased mice, images were captured from sites free of lymphocytic infiltrates since these are devoid of both acini and MECs. To conduct an unbiased determination of MEC size, we used ImageJ software (version 1.50e, http://imagej.nih.gov/ij) to analyze fragment size using standard procedures and the following parameters. First, background fluorescence was removed using a rolling ball diameter of 10 pixels. Next, the pixel intensity threshold was set to a minimum of 8 to create a masked image of the fluorescence. Finally using the “Analyze Particles” function, area measurements in µm^2^ were taken of all particle fragments within the masked image with parameters size >20 µm^2^ and circularity = 0.00–0.75 (MECs should be more linear than circular).

### SDS-PAGE and western blotting

Excised lacrimal glands and sorted cells were homogenized for 20 minutes in 0.4 mL ice-cold radio-immunoprecipitation assay (RIPA) buffer (10 mM Tris-HCl pH 7.4, 150 mM NaCl, 1 mM EDTA, 1% Triton X-100, 0.1% sodium deoxycholate, and 0.1% SDS supplemented with protease inhibitors). Following homogenization tissue lysates were centrifuged at 20,000x*g* for 30 minutes and the protein supernatant was collected. Proteins were separated by SDS-PAGE on NuPage 4–12% Bis-Tris gels in MOPS-SDS buffer (Invitrogen, Carlsbad, CA). Protein gels were transferred to PVDF membranes using NuPage transfer buffer (Invitrogen) for immunoblotting.

After transfer, PVDF membranes were stained with LI-COR REVERT Total Protein Stain, per the manufacturer’s instructions, prior to being blocked using Odyssey blocking buffer (LI-COR Biosciences, Lincoln, NE) for 1 hour at room temperature. Membranes were incubated overnight at 4 °C with the appropriate primary antibody (Table 1) diluted in blocking buffer. Following washing with Tris-buffered saline + tween-20 (TBS; 50 mM Tris-HCl, 150 mM NaCl, 0.1% tween-20; pH 7.6) membranes were then incubated for 1 hour at room temperature with the appropriate secondary antibodies followed by detection on a LI-COR Odyssey Infrared Imager. Staining in each lane for REVERT total protein stain, and band intensity for immunoblotting was quantified using the LI-COR Image Studio software (v.4.0). Western blot band quantifications were then normalized to the total amount of protein in each lane.

### RNA Extraction and Reverse Transcription (RT)-PCR

RNA was extracted from excised lacrimal glands using the miRNeasy isolation kit (Qiagen, Valencia, CA), using the manufacturer’s protocol. Briefly, the tissue was homogenized in QIAzol lysis buffer and incubated for 5 minutes at room temperature. Chloroform was then added to the homogenate, followed by centrifugation at 12,000 × g at 4 °C. Next, the aqueous layer was mixed with 1.5 volumes of absolute ethanol and run through the MiniElute spin columns. Buffers RWT, RPE (both provided in kit), and 80% ethanol were then sequentially run through to wash the column. Finally, 20 µL of RNAse-free distilled water was used to elute RNA from the column. RNA samples were stored at −80 °C until downstream use.

The concentration and purity of RNA was first assessed using a NanoDrop 1000 (ThermoFisher Scientific, Waltham, MA). Purified total RNA (50–250 ng) was used for reverse transcription and PCR amplification with the OneStep RT-PCR Kit (Qiagen) with primers designed using NCBI/ Primer-BLAST (Table 2) in a 2720 Thermal Cycler (Applied Biosystems, Foster City, CA). The reverse transcription reaction was conducted at 50 °C for 30 minutes followed by PCR according to the manufacturer’s instructions. The cycling conditions were 15 minutes hot start at 95 °C, followed by 30 to 40 cycles of denaturation for 40 seconds at 94 °C, annealing for 40 seconds at 52 °C, and extension for 1 minute at 72 °C. A final extension at 72 °C for 10 minutes was performed and the amplification products were separated by electrophoresis on a 1.5% agarose gel and visualized by UV light after ethidium bromide staining. All PCR bands were normalized to the internal GAPDH control for each sample.

**Table 2:** Primers used for semi-quantitative RT-PCR.

Target	Primers		Amplicon Size (bp)
***Mouse:***			
α-SMA	F:	5′-CTGACAGAGGCACCACTGAA-3′	160
	R:	5′-CATCTCCAGAGTCCAGCACA-3′	
Calponin	F:	5′-CCTTCCCAGGATTGACCCAC-3′	206
	R:	5′-AAACCCCCACAAAACCCCTT-3′	
GAPDH	F:	5′-GGTGAAGGTCGGTGTGAACG-3′	233
	R:	5′-CTCGCTCCTGGAAGATGGTG-3′	
Oxytocin Receptor	F:	5′-GATGTCGCTCGACCGCTG-3′	240
	R:	5′-CGGTACAATGTAGACGGCGA-3′	
***Human:***			
Oxytocin Receptor	F:	5′-CTGGACGCCTTTCTTCTTC-3′	230
	R:	5′-GACAAAGGAGGACGAGTTG-3′	

### Measurement of MEC contraction

MEC contraction was measured using the protocol described by Haaksma *et al*.^27^ with minor modifications. Briefly, lacrimal glands were dissected and the thin capsule, which covers the gland’s surface, was removed with forceps. Lacrimal gland lobes were separated and rinsed in buffer-I (136 mM NaCl, 1.2 mM NaH_2_PO_4_, 1.2 mM MgSO_4_, 5 mM KCl, 1.7 mM CaCl_2_, 1.1 mM glucose, 0.03 mM Na_2_EDTA, 10 mM HEPES, pH 7.4) and then incubated in the same buffer for 10 min at 37 °C. Oxytocin (0–3251; Sigma-Aldrich) dissolved in buffer-I was then added to the dishes to a final concentration of 5 nM for 2 min at 37 °C. Immediately following incubation the tissue was fixed in 4% formaldehyde (diluted in buffer-I), washed in PBST (PBS + 0.05% tween-20) and stained with rhodamine phalloidin (to visualize F-actin) and DAPI (to visualize nuclei). Lobes were mounted and analyzed using a Zeiss-780 confocal laser-scanning microscope. Images were processed using the IMARIS software. Acinar size (area, perimeter, and Feret’s diameter, as depicted in Fig. 7E) was measured using ImageJ software. Forty to fifty acini from 3 mice were randomly measured per group.

### Statistical analyses and data presentation

All statistical analyses were performed using GraphPad Prism Software (version 7.0; San Diego, CA) and data are presented as means ± standard error of the mean (SEM). Data consisting of 2 groups were analyzed using a 2-tailed unpaired Student’s *t*-test or the Mann-Whitney U test, for non-normally distributed data, with significant results being considered with p-value < 0.05.

### Data Availability

All data generated or analyzed during this study are included in this published article or the supplementary information file.

## Electronic supplementary material

Supplemental Material
