# Supplementary material for: Myoepithelial cell-driven acini contraction in response to oxytocin receptor stimulation is impaired in lacrimal glands of Sjögren’s syndrome animal models
Source: Sci Rep. 2018 Jul 2;8:9919. doi: 10.1038/s41598-018-28227-x (PMC6028591; doi:10.1038/s41598-018-28227-x)

Myoepithelial cell-driven acini contraction in response to oxytocin receptor stimulation is impaired in lacrimal glands of Sjögren's syndrome animal models

Dillon Hawley[1], Xin Tang[2], Tatiana Zyrianova[2], Mihir Shah[3], Srikanth Janga[3], Alexandra Letourneau[1], Martin Schicht[4], Friedrich Paulsen[4], Sarah Hamm-Alvarez[3,5], Helen P. Makarenkova[2], and Driss Zoukhri[1,6*]

[1]Depratement of Comprehensive Care, Tufts University School of Dental Medicine, Boston, MA, USA

[2]Department of Molecular Medicine, The Scripps Research Institute, La Jolla, CA, USA

[3]Department of Ophthalmology, USC Roski Eye Institute, Keck School of Medicine of University of Southern California, Los Angeles, CA, USA

[4]Department of Anatomy II, Friedrich Alexander University Erlangen-Nürnberg, Erlangen, Germany

[5]University of Southern California School of Pharmacy, Los Angeles, CA, USA

[6]Department of Ophthalmology, Tufts University School of Medicine, Boston, MA, USA

Driss Zoukhri: driss.zoukhri@tufts.edu (*corresponding author)

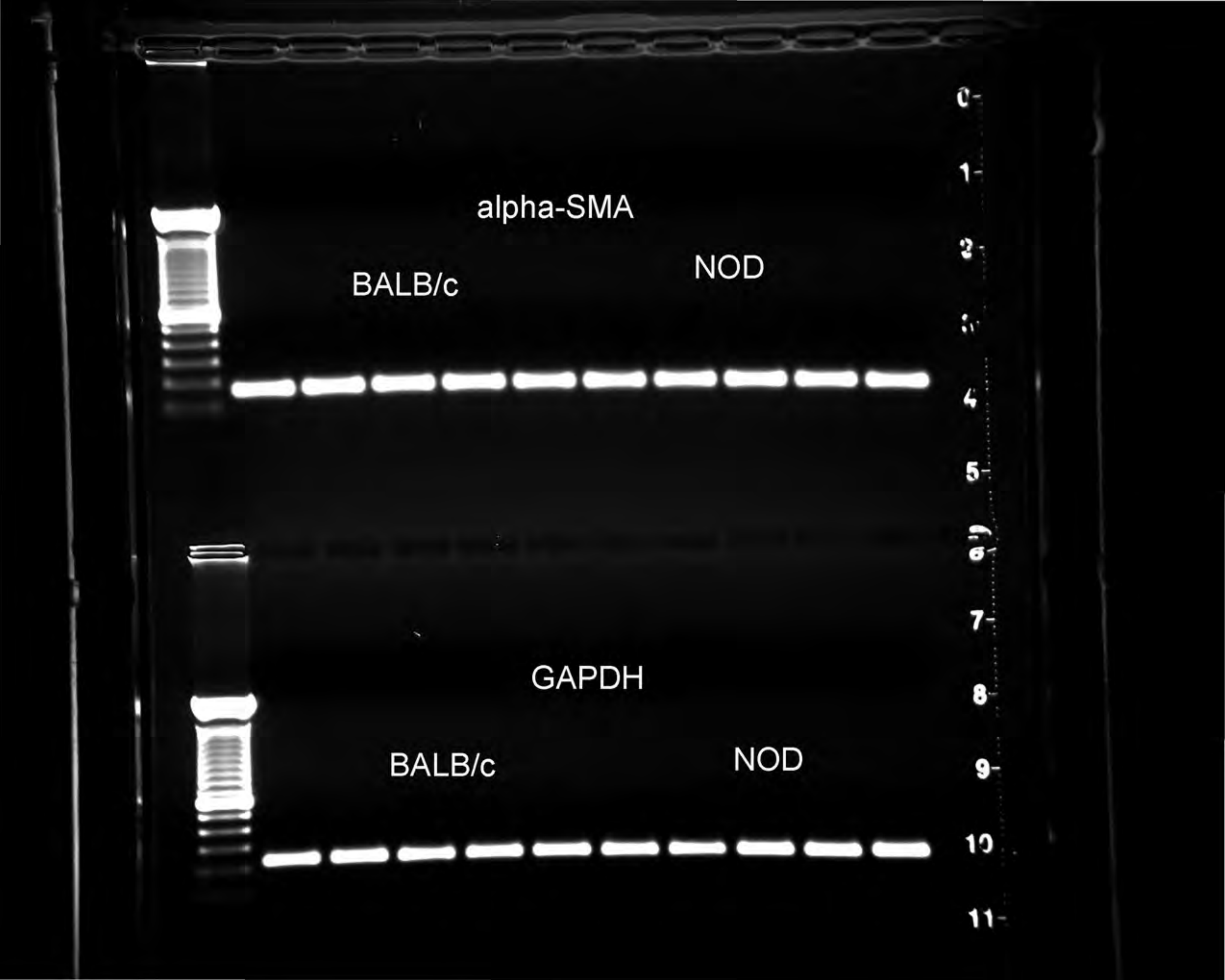
alpha-SMA
BALB/c
NOD
GAPDH
BALB/c
NOD

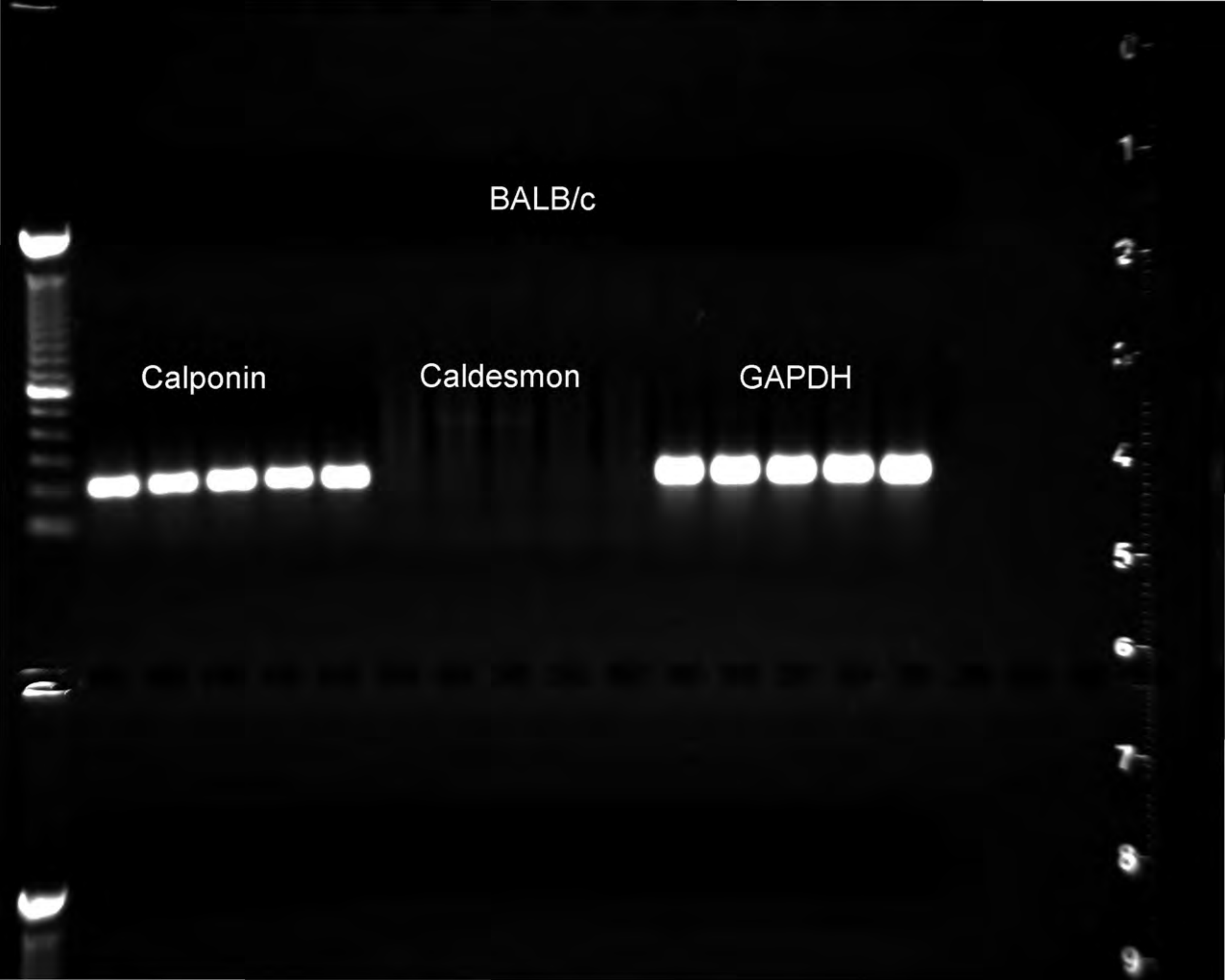
BALB/c
Calponin
Caldesmon
GAPDH

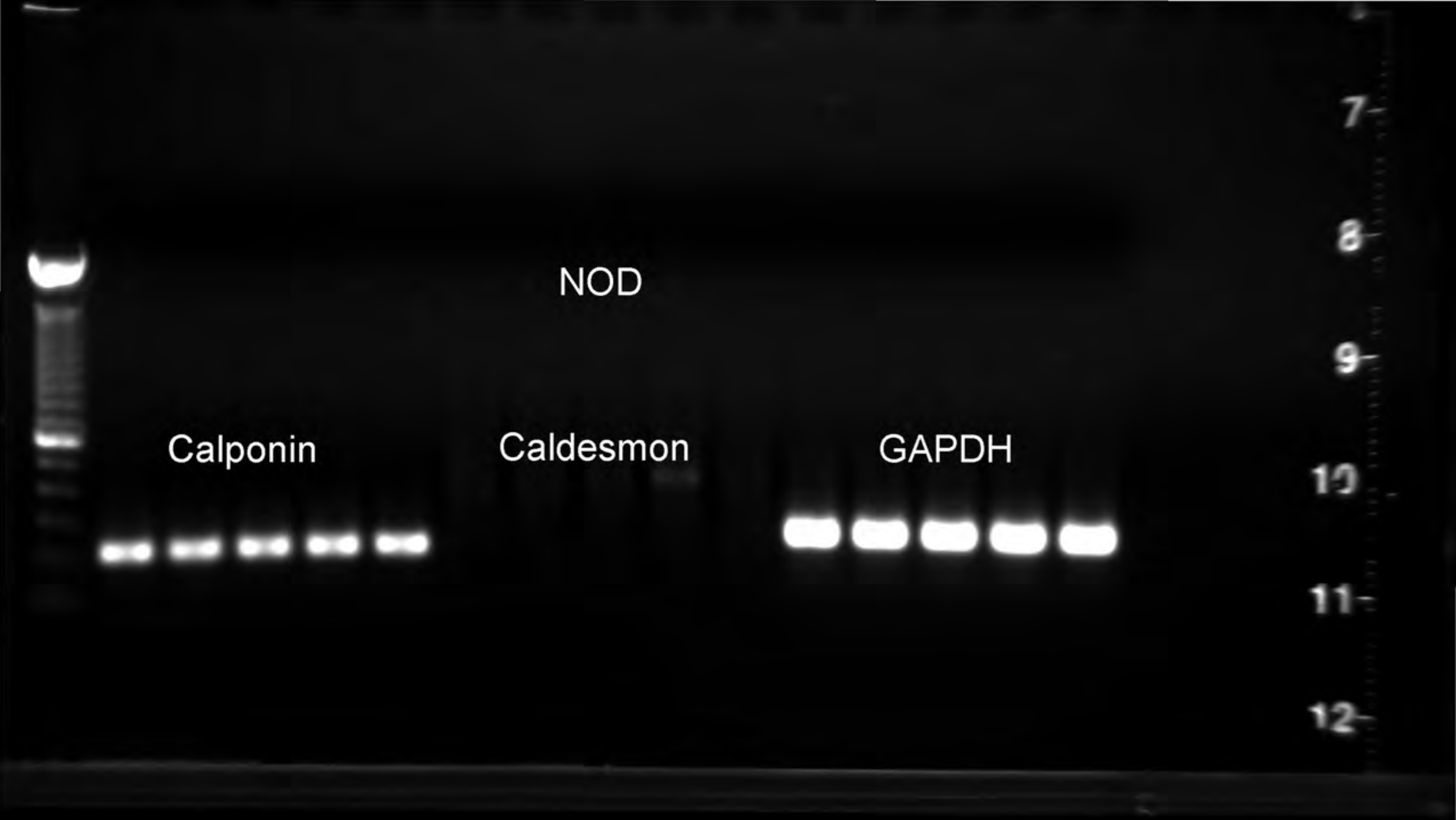
NOD
Calponin
Caldesmon
GAPDH
7
8
9
10
11
12

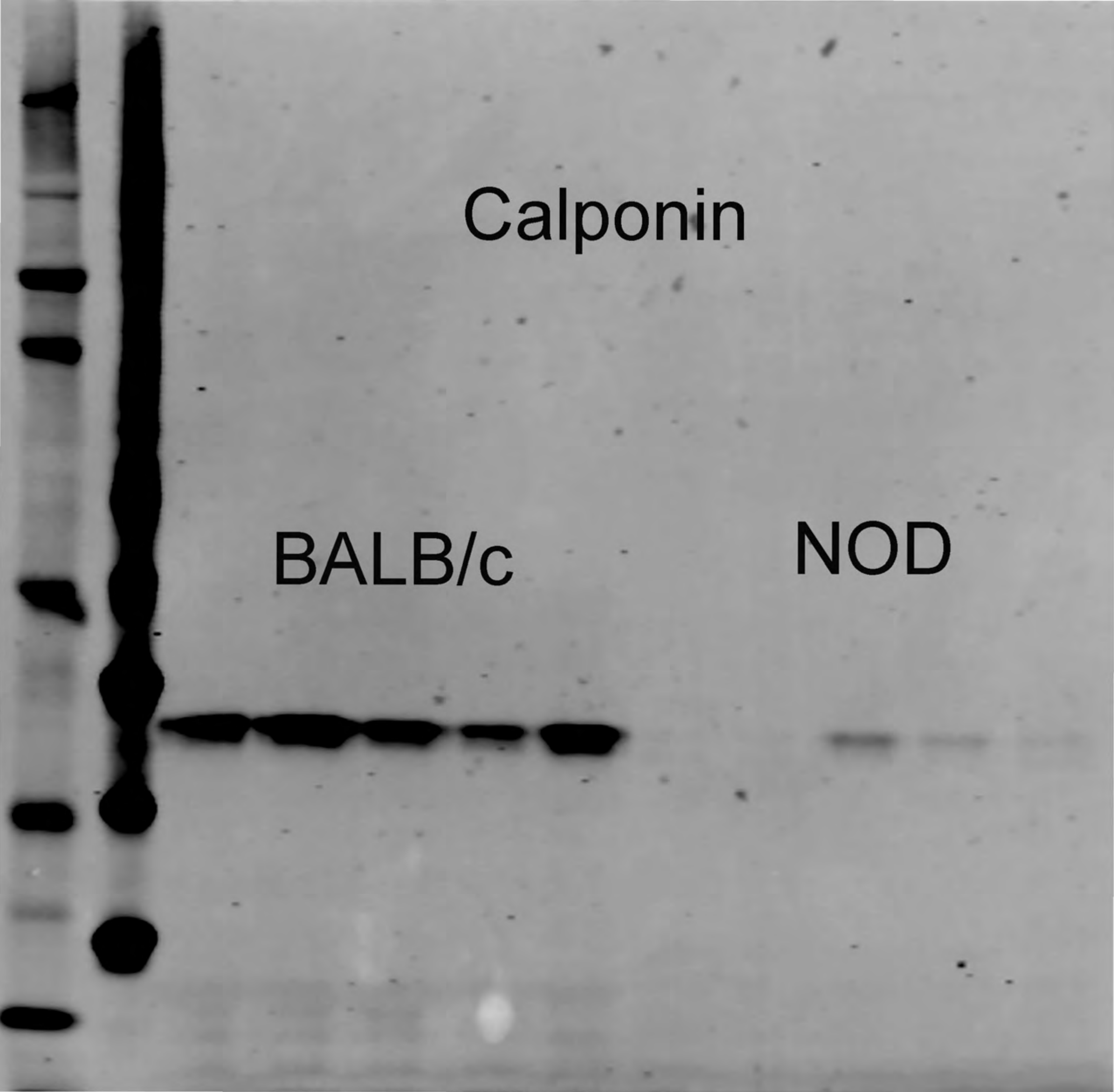
Calponin
BALB/c
NOD

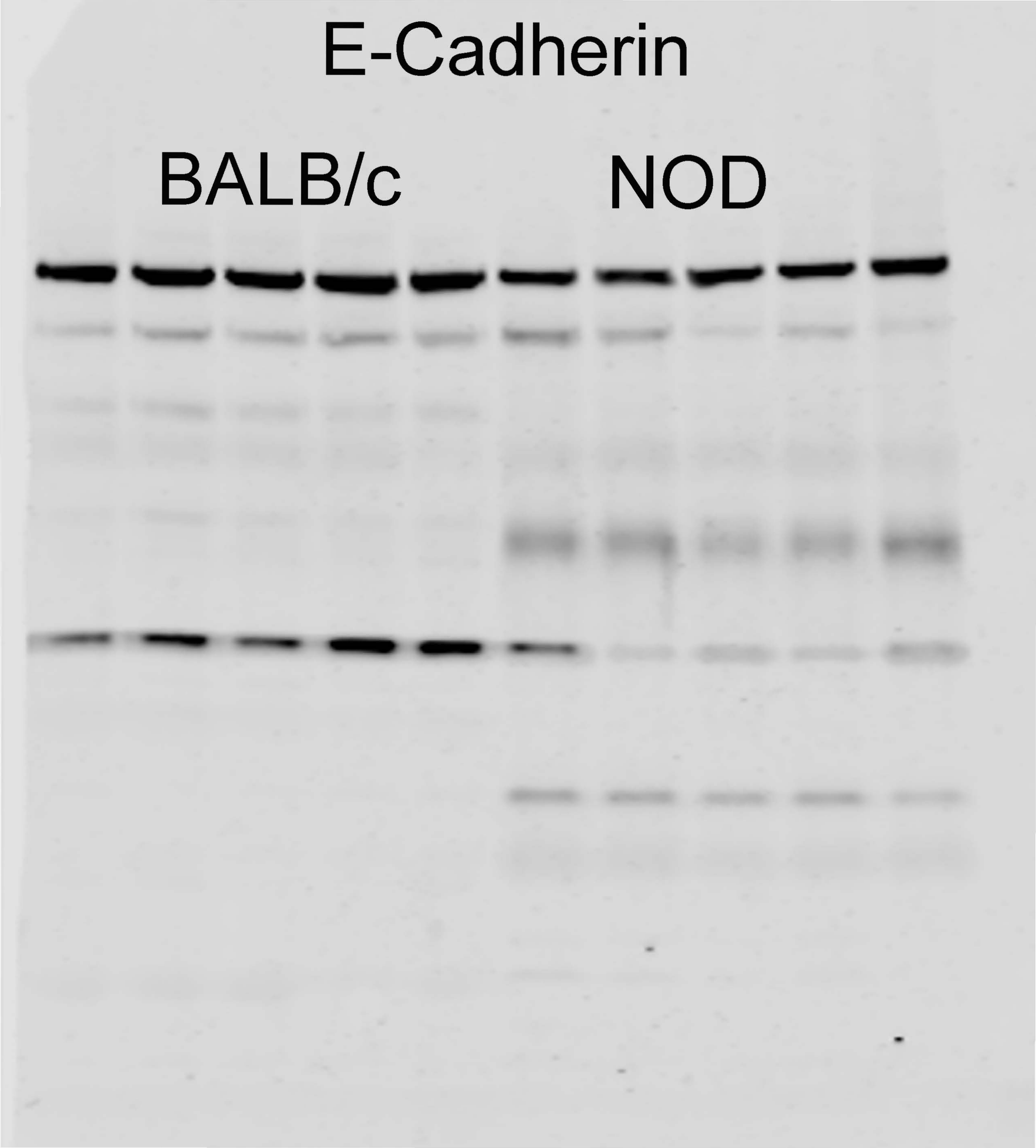
E-Cadherin
BALB/c
NOD

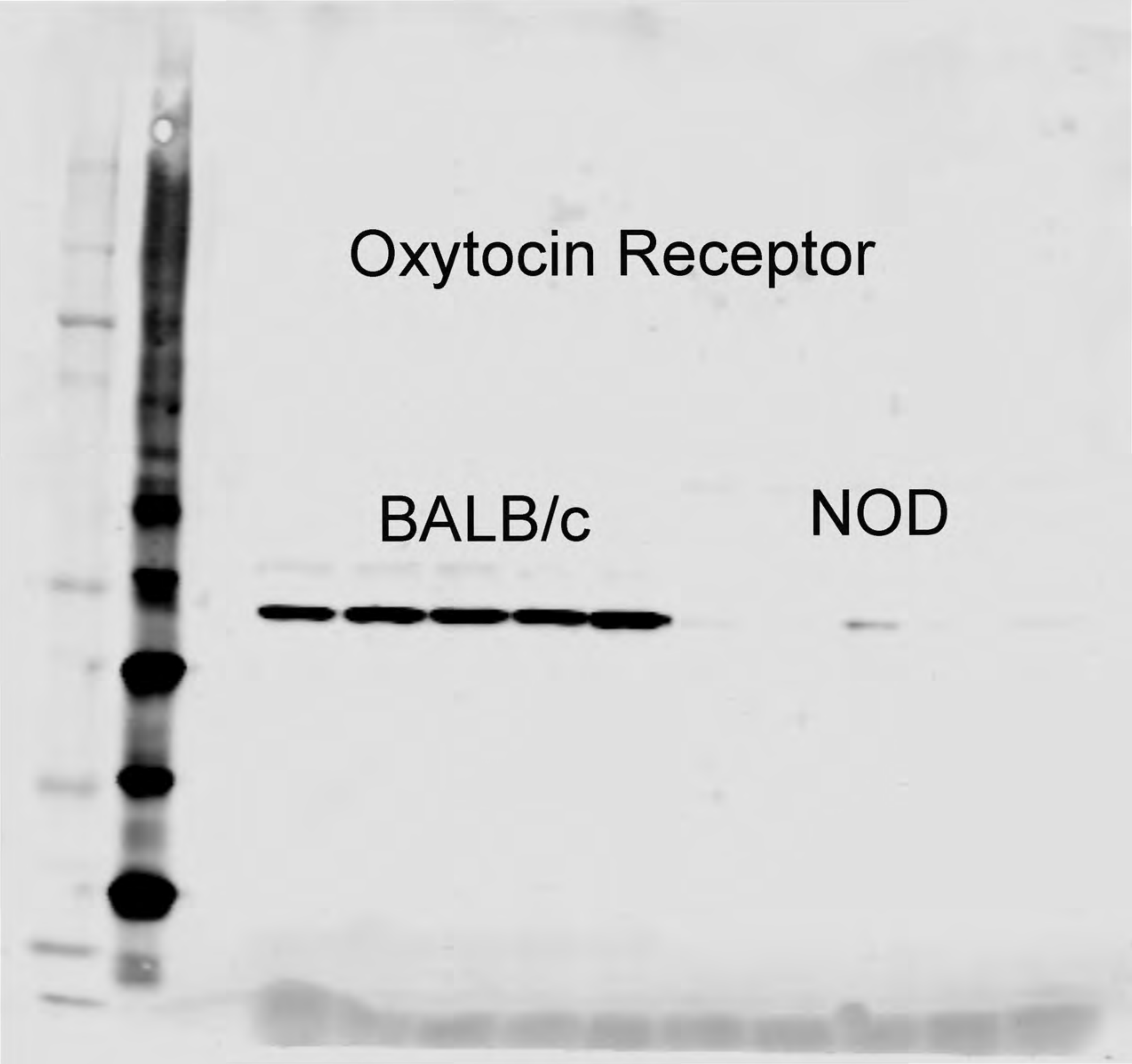
Oxytocin Receptor
BALB/c
NOD

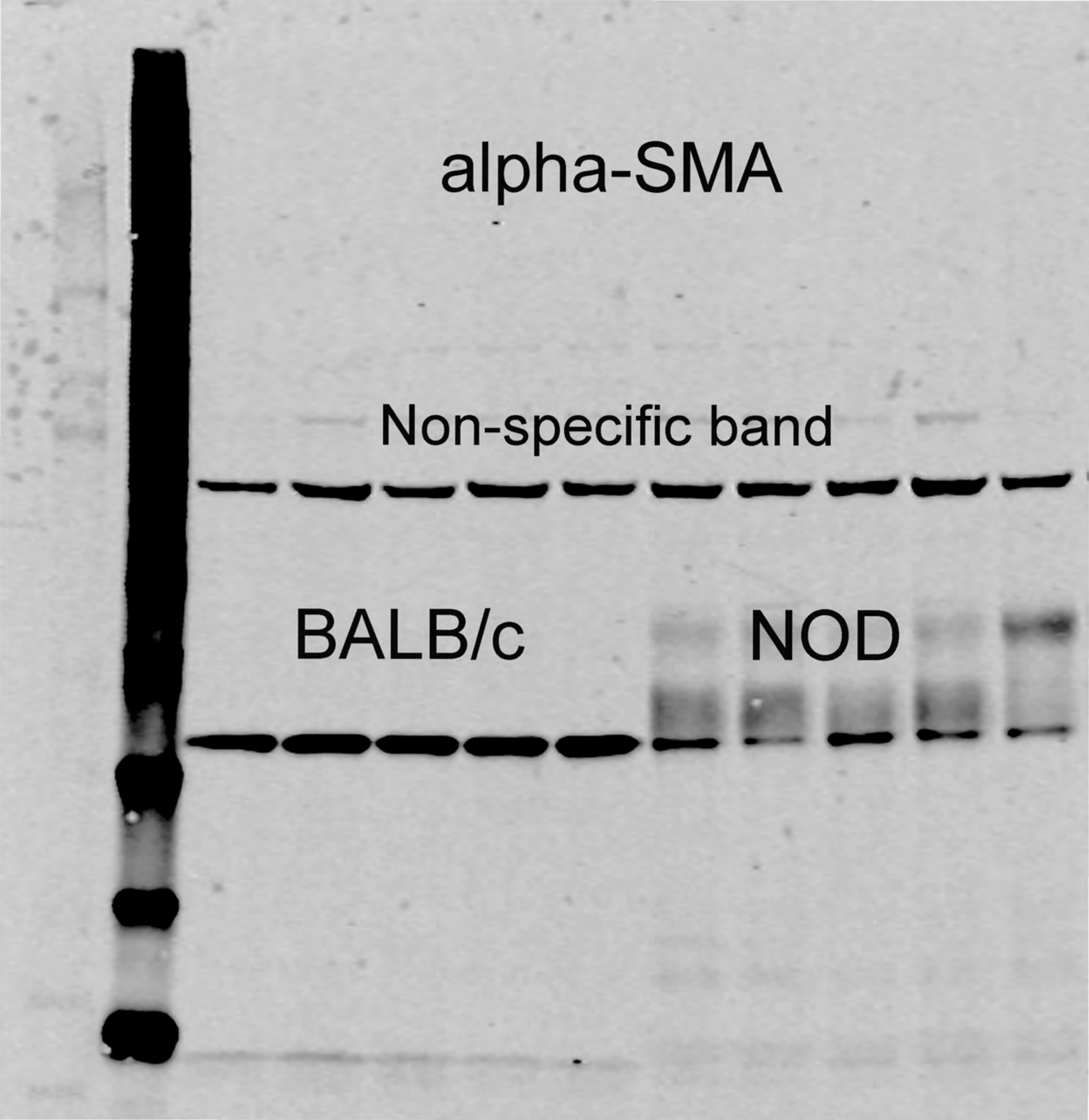

alpha-SMA
Non-specific band
BALB/c
NOD

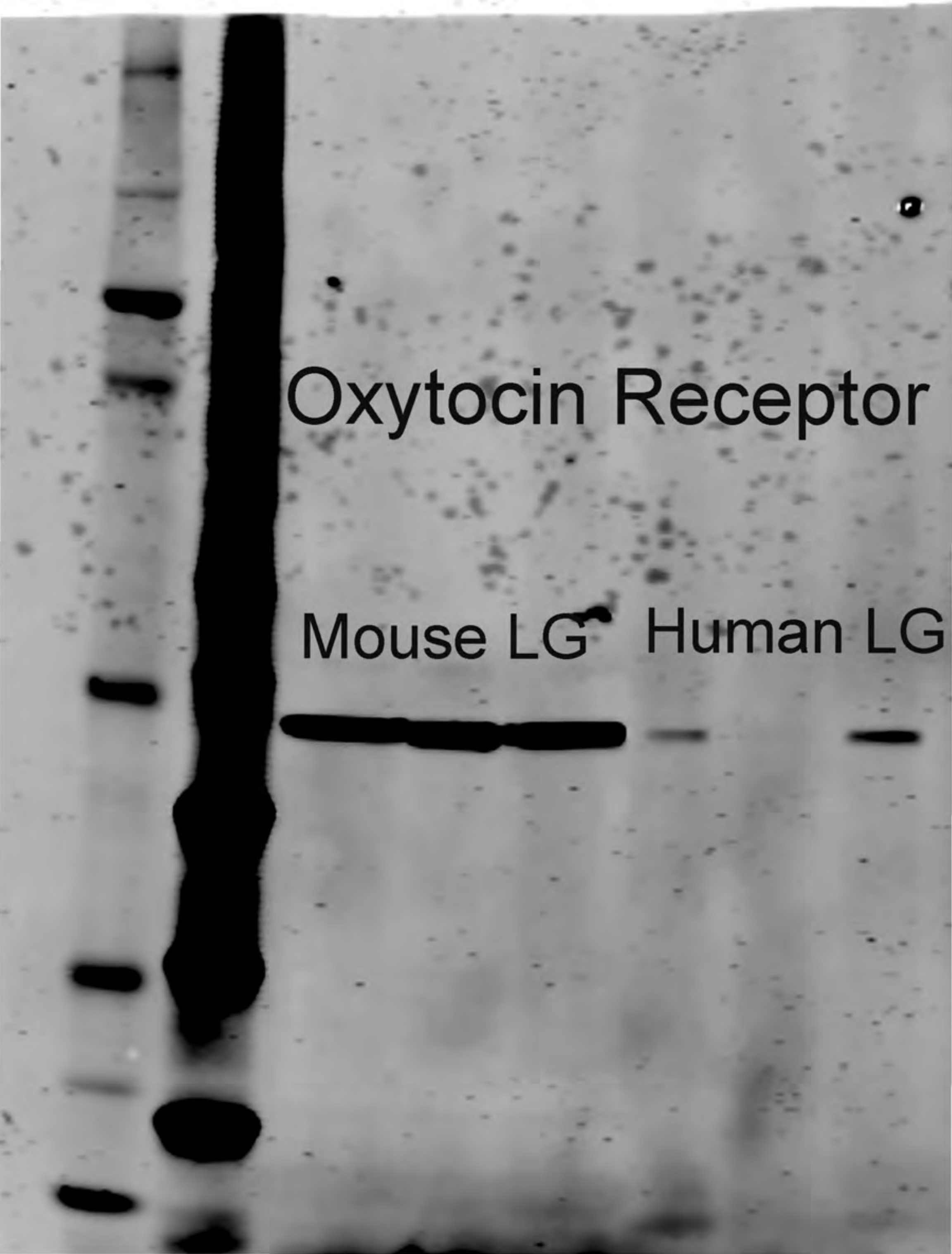
Oxytocin Receptor
Mouse LG
Human LG

Fig 1D

	BALB/c	NOD
**AVG**	79.92	54.24
**STDEV**	11.67	12.15
**% Decrease**		32.13

Fig 1F

	MRL +/+	MRL/lpr
**AVG**	69.10	58.87
**STDEV**	9.29	15.53
**% Decrease**		14.82

Fig 2A

			BALB/C		
	**50**	**100**	**150**	**200**	**More**
**AVG**	45.96	28.90	12.77	6.23	6.15
**STDEV**	3.53	2.28	1.54	2.72	2.12
			**NOD**		
	**50**	**100**	**150**	**200**	**More**
**AVG**	64.54	25.04	6.17	2.37	1.87
**STDEV**	8.67	3.06	2.25	1.59	2.49
**% Decrease**	-40.44	13.34	51.69	61.92	69.51

Fig 2B

			MRL +/+		
	**50**	**100**	**150**	**200**	**More**
**AVG**	52.58	29.01	9.78	4.64	3.98
**STDEV**	3.67	1.03	0.85	1.03	1.57
			**MRL/lpr**		
	**50**	**100**	**150**	**200**	**More**
**AVG**	61.83	26.19	6.73	2.37	2.88
**STDEV**	10.89	4.47	2.62	1.58	2.80
**% Decrease**	-17.58	9.74	31.23	48.91	27.52

Fig 3B

	BALB/c		NOD		
	**AVG**	**STDEV**	**AVG**	**STDEV**	**% Decrease**
**α-SMA**	22.80	2.77	9.20	2.49	59.65
**Calponin**	29.71	13.56	1.54	2.10	94.82
**Caldesmon**	1.47	0.24	3.93	2.64	-166.99
**E-Cadherin**	29.98	2.48	24.11	5.16	19.57

Fig 3D

	BALB/c		NOD		
	AVG	STDEV	AVG	STDEV	% Decrease
α-SMA	1.19	0.04	1.13	0.07	4.87
Calponin	0.93	0.05	0.89	0.01	4.29

Fig 6F

	BALB/c		NOD	
	Vehicle	Oxytocin	Vehicle	Oxytocin
AVG	3287.60	2617.70	3823.85	3599.50
STDEV	124.74	91.42	136.49	161.03
% Decrease to Vehicle		20.38		5.87
% Increase to Control			16.31	

Fig 6G

	BALB/c		NOD	
	Vehicle	Oxytocin	Vehicle	Oxytocin
AVG	214.96	191.53	234.14	222.33
STDEV	3.87	3.75	4.28	4.40
% Decrease to Vehicle		10.90		5.05
% Increase to Control			8.92	

Fig 6H

	BALB/c		NOD	
	Vehicle	Oxytocin	Vehicle	Oxytocin
AVG	78.58	68.08	85.53	80.77
STDEV	1.49	1.34	1.73	8.12
% Decrease to Vehicle		13.36		5.57
% Increase to Control			8.85	

Fig 7B

	BALB/c		NOD		
	AVG	STDEV	AVG	STDEV	% Decrease
OXTR	11.81	3.75	0.15	0.20	98.73

Supplemental FACS plot of sorted GFP+ myoepithelial cells.

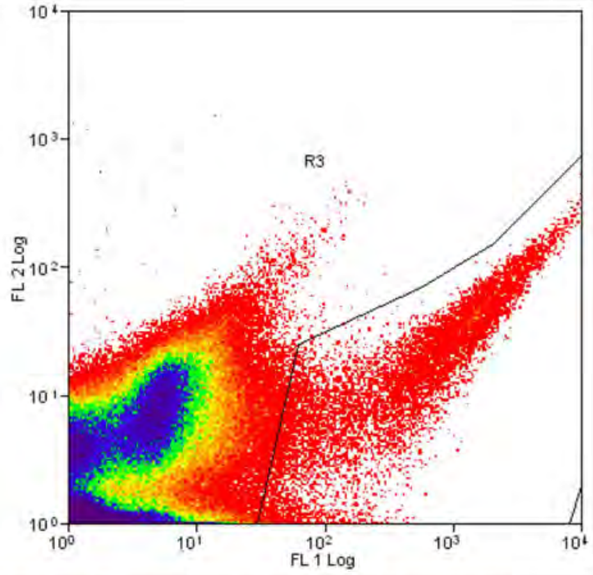

Region	Count	% Hist	% All	Mean
Total	284156	100.00	56.83	41.44, 6.81
R3	11700	4.12	2.34	887.77, 25.58

Supplemental figures showing lacrimal gland blood vessel staining, using an antibody against PECAM-1, in BALB/c and NOD mice (Fig 1). The percent area covered by blood vessels was calculated using ImageJ software (Fig 2). Figure 3 shows a lower magnification of PECAM-1 stained blood vessels from BALB/c and NOD mice lacrimal glands.

Figure 1

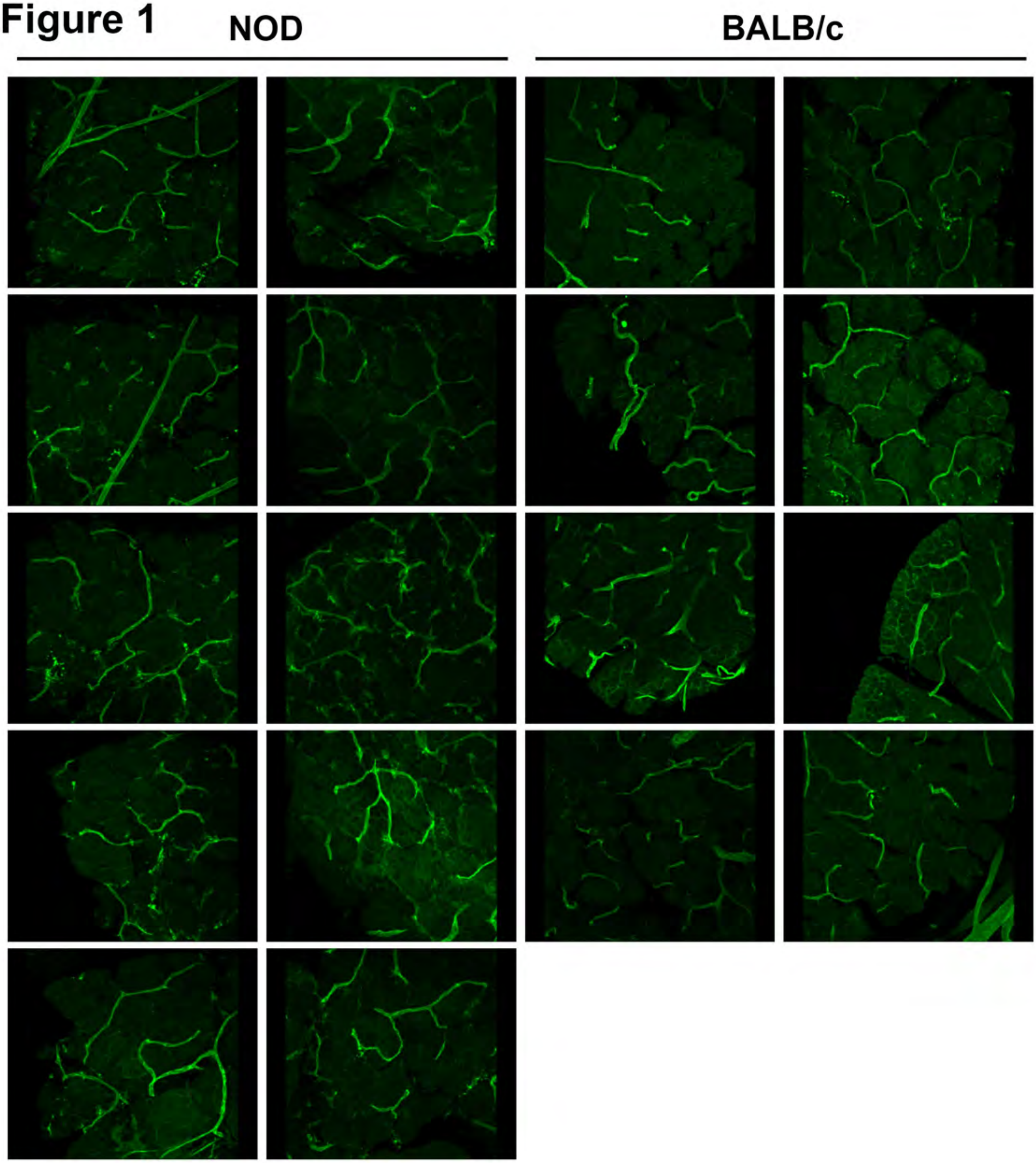

Figure 2

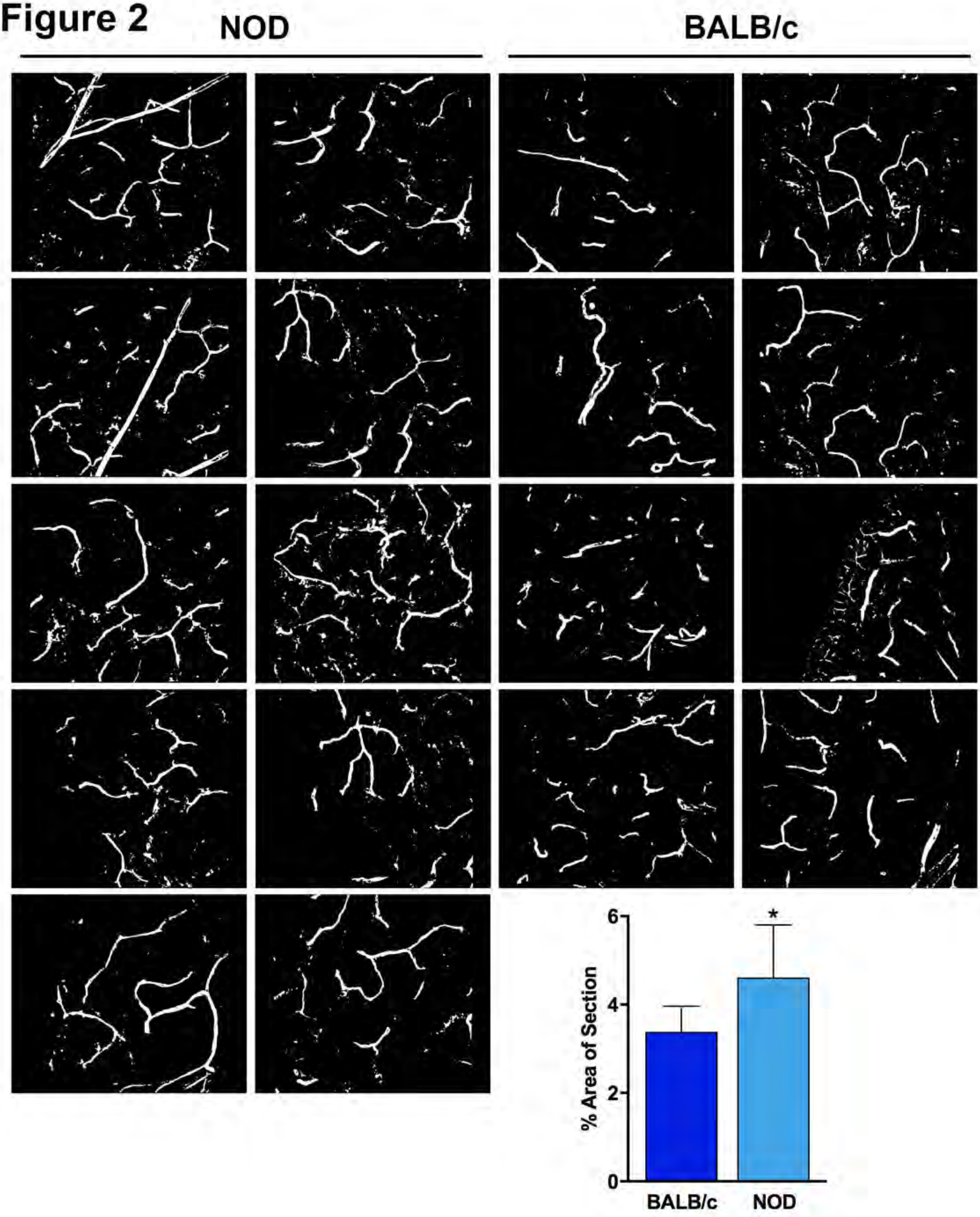

Figure 3

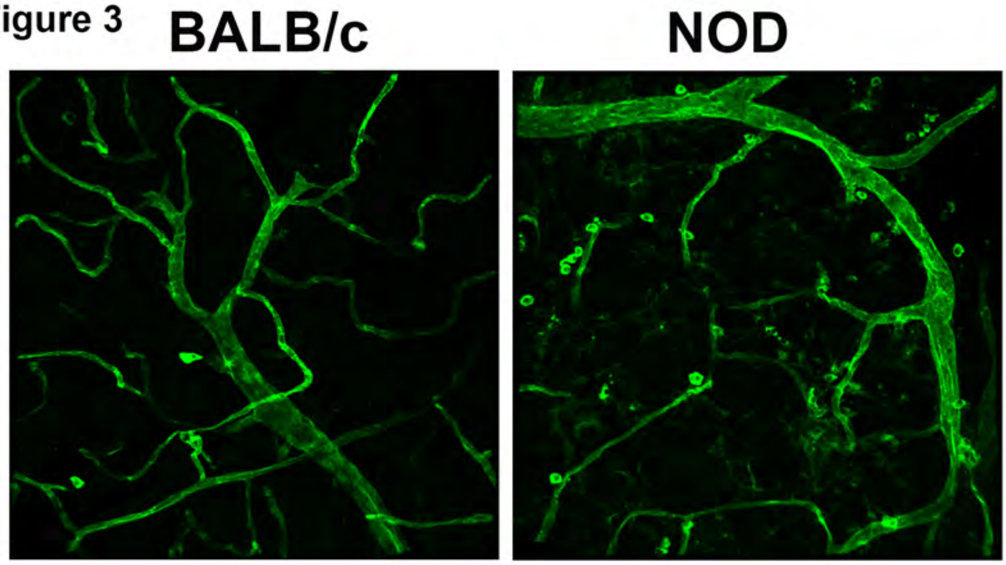

Supplement: Supplementary file 1 — Supplemental Material [file 41598_2018_28227_MOESM1_ESM.pdf]
